# Digital health engagement profiles and physical activity guideline adherence among adults in the United States: a latent class analysis

**DOI:** 10.3389/fphys.2026.1901594

**Published:** 2026-09-16

**Authors:** Heejun Kim, Ju-Pil Choe, Minsoo Kang

**Affiliations:** Health and Sport Analytics Laboratory, Department of Kinesiology and Sport, The University of Mississippi, University, MS, United States

**Keywords:** digital health, Health Information National Trends Survey, latent class analysis, mobile health, physical activity guidelines, wearable devices

## Abstract

**Background:**

Physical activity is a key modifiable factor for preventing chronic disease, and digital health technologies are increasingly used to support physical activity. Prior population-based studies have typically examined single digital health behaviors, particularly wearable use, rather than combinations of behaviors that may cluster within individuals.

**Methods:**

We analyzed 2024 Health Information National Trends Survey (HINTS 7) data from U.S. adults. Latent class analysis was applied to 17 binary indicators across five digital health domains among 5,804 respondents with complete coded digital health indicator data. Associations between profile membership and adherence to the Physical Activity Guidelines for Americans were examined using survey-weighted logistic regression among 4,930 respondents with complete physical activity outcome and covariate data. The primary outcome was concurrent adherence to both aerobic and muscle-strengthening guidelines; aerobic and muscle-strengthening adherence were examined separately as secondary outcomes.

**Results:**

Four profiles were identified: Broad Multidomain Engagement (32.0%), Wearable-Centered Engagement (11.8%), Portal-Centered Engagement (34.3%), and Limited Engagement (21.9%; reference), with high classification quality (entropy = 0.911). Relative to Limited Engagement, the Broad Multidomain Engagement (adjusted odds ratio [aOR] = 2.79, 95% CI: 1.81–4.30) and Wearable-Centered Engagement (aOR = 2.13, 95% CI: 1.42–3.19) profiles had higher odds of meeting both guidelines concurrently, whereas Portal-Centered Engagement membership was not significantly associated with concurrent adherence (aOR = 1.36, 95% CI: 0.97–1.89). In the primary complete-case models, only Broad Multidomain Engagement was associated with aerobic adherence (aOR = 1.64, 95% CI: 1.17–2.29), while all three digitally engaged profiles were associated with muscle-strengthening adherence (aOR range 1.58–2.58).

**Conclusions:**

Digital health engagement among U.S. adults is organized into distinct multi-domain profiles that are differentially associated with physical activity guideline adherence. These findings support person-centered approaches to studying digital health behavior and may inform future profile-guided physical activity support, although longitudinal and intervention studies are needed before causal or adaptive-intervention claims can be made.

## 1 Introduction

Physical activity is essential for preventing chronic disease and premature mortality (Lee et al., 2012; Warburton and Bredin, 2017), yet a substantial proportion of U.S. adults do not meet the aerobic and muscle-strengthening recommendations set out in the 2018 Physical Activity Guidelines for Americans (U.S. Department of Health and Human Services, 2018). At the same time, engagement with digital health technologies, including wearable devices, mobile health applications, patient portals, social media, and telehealth, has become increasingly embedded in everyday health behavior. Population-based evidence suggests that digital health engagement is heterogeneous across behaviors: online health information seeking is relatively common, whereas health app use, digital tracking, provider communication, data sharing, and sharing health information on social media occur less consistently and vary by sociodemographic characteristics (Ratcliff et al., 2021). Because these technologies are widely promoted as tools for self-monitoring, health communication, and physical activity promotion, understanding how digital health engagement relates to guideline adherence at the population level has become an important public health question.

Digital health engagement encompasses multiple functions and cannot be reduced to the use of a single technology. A wearable device, for example, may support both self-monitoring and the sharing of personal health data, whereas a patient portal may support both access to clinical information and communication with health care providers. A functional perspective therefore describes digital health engagement in terms of what individuals do rather than which device they use. Survey research has shown that the purposes for which people use digital health services can be empirically grouped into broader functions from a user perspective (Grimm et al., 2023). Established taxonomies follow the same functional logic. The World Health Organization’s Classification of Digital Interventions, Services and Applications in Health, for example, distinguishes personal health tracking, on-demand look-up of health and health service information, person-to-person peer communication, and telemedicine consultation, each of which may be delivered through more than one technology (World Health Organization, 2023). Informed by these perspectives rather than derived from any single classification, we grouped the available HINTS 7 indicators into five domains labeled by their primary technological or service context: wearable device use, mobile health applications and data sharing, online health information and patient portal engagement, social media health engagement, and telehealth use.

The Health Information National Trends Survey (HINTS), a nationally representative survey administered by the National Cancer Institute, has been used for more than a decade to study physical activity and health communication in the U.S. population (Oh et al., 2010; Valle et al., 2015), making it a well-suited platform for addressing this question. Its breadth has supported population-level description of several forms of digital health engagement, including online health information seeking, patient-provider communication, health application use, and digital tracking (Ratcliff et al., 2021). Early HINTS work in this area showed that using the internet for diet, weight, and physical activity information was associated with physical activity-related behaviors, supporting the relevance of online health engagement for behavioral outcomes (McCully et al., 2013). Subsequent studies linking digital health engagement to physical activity, however, have concentrated on individual technologies, particularly wearable devices and mobile health applications. Wearable device users have been shown to have higher odds of meeting physical activity recommendations than non-users, with adjusted odds ratios of 1.53 in 2019 HINTS data (Xie et al., 2020) and 1.84 in 2022 HINTS data (Lee et al., 2025), consistent with systematic review evidence suggesting that consumer-based wearable activity trackers can increase physical activity participation when used as part of behavioral interventions (Brickwood et al., 2019). Wearable use has also been examined in relation to self-efficacy for managing health, predictors of adoption, and inequities in use among adults with or at risk for cardiovascular disease (Chandrasekaran et al., 2020; Dhingra et al., 2023; Xie et al., 2021). This focus on wearable-based evidence leaves unclear how other digital health behaviors co-occur with wearable use and whether broader patterns of engagement are associated with physical activity guideline adherence.

Despite this progress, three gaps remain. First, digital health engagement has often been treated as a single behavior, most commonly wearable use operationalized as a binary yes/no indicator, even though contemporary users may engage across the five domains described above. Patient portals support access to personal clinical information, appointment and care management, and electronic patient-provider communication (Carini et al., 2021). Social media health engagement represents a distinct but complementary form of digital health behavior when platforms are used for identifiable health-related purposes, including seeking and sharing health information, exchanging social support, and communicating health experiences with others (Moorhead et al., 2013). In a systematic review of social media use for health purposes, Chen and Wang (2021) identified three forms of public use for health purposes: seeking and sharing health information, exchanging social support in online communities, and tracking and sharing personal health status or activities. The third form, tracking and sharing personal health status or activities, includes documenting and sharing fitness activities such as walking and cycling. These functions provided the conceptual basis for including social media indicators in the present study. Second, prior work has generally emphasized whether or how much a given technology is used rather than which combinations of digital health behaviors co-occur. HINTS-based work on mobile health use has shown that adults can be characterized by distinct patterns of device ownership and health or behavioral tracking, suggesting that digital health behavior is better understood as patterned engagement than simple use versus nonuse (Rising et al., 2020). Third, physical activity has often been treated as a single outcome, with less attention to whether digital health engagement relates differently to aerobic and muscle-strengthening adherence. This distinction is important because these activity types differ in how they are performed, monitored, and potentially supported by digital tools. Consequently, it remains unclear which digital health behavior profiles exist among U.S. adults and whether these profiles are differentially associated with meeting physical activity guidelines.

Latent class analysis (LCA) is well suited to this question because it estimates unobserved subgroups defined by patterns of responses across multiple indicators rather than treating each digital health behavior as an independent exposure. In this context, LCA can distinguish profiles centered on wearable self-monitoring, patient-portal engagement, telehealth, or broader multi-channel use. This person-centered approach aligns with the substantive question of whether configurations of digital health behavior, not simply the use of any single tool, are associated with physical activity guideline adherence. Latent class and related latent class growth approaches have already been applied productively within physical activity research to identify behavioral subgroups and link them to health outcomes (Choe et al., 2026; Kim et al., 2016a, Kim et al., 2016b).

To address these gaps, the present study applied a person-centered analysis to 17 digital health behavior indicators from HINTS 7, organized into the five domains described above. We conducted LCA to identify digital health behavior profiles and then used survey-weighted regression to examine associations between profile membership and physical activity guideline adherence. Two research questions guided the study: (RQ1) What latent profiles of digital health behaviors can be identified among U.S. adults? (RQ2) How are these profiles associated with concurrent adherence to both the aerobic and muscle-strengthening guidelines and with adherence to each guideline separately? This framework moves beyond single-behavior and binary measures of digital health use toward a person-centered account of how combined patterns of digital health behavior relate to physical activity adherence.

## 2 Materials and methods

### 2.1 Study design and analytic samples

Data were drawn from the seventh cycle of the Health Information National Trends Survey (HINTS 7), a nationally representative cross-sectional survey of civilian non-institutionalized U.S. adults aged 18 years or older administered by the National Cancer Institute. Data were collected between March and September 2024 using a multi-mode protocol with web response and paper backup; the final sample comprised 7,278 respondents (American Association for Public Opinion Research [AAPOR] Response Rate 4: 27.3%). Detailed sampling, data collection, and weighting procedures, including the stratified address-based design and raking calibration to U.S. population control totals, are described in the HINTS 7 Methodology Report (National Cancer Institute, 2025). Because HINTS is a publicly available, de-identified secondary dataset, this study was exempt from institutional review board review.

Two nested analytic samples were used (**Figure 1**). The LCA sample included 5,804 respondents with complete data on the 18 candidate digital health behavior indicators. The regression sample included 4,930 of these respondents who also had complete data on the physical activity outcomes and all covariates. For regression analyses, each respondent’s class membership was assigned based on the final four-class LCA solution using their most-likely class.

**Figure 1:**
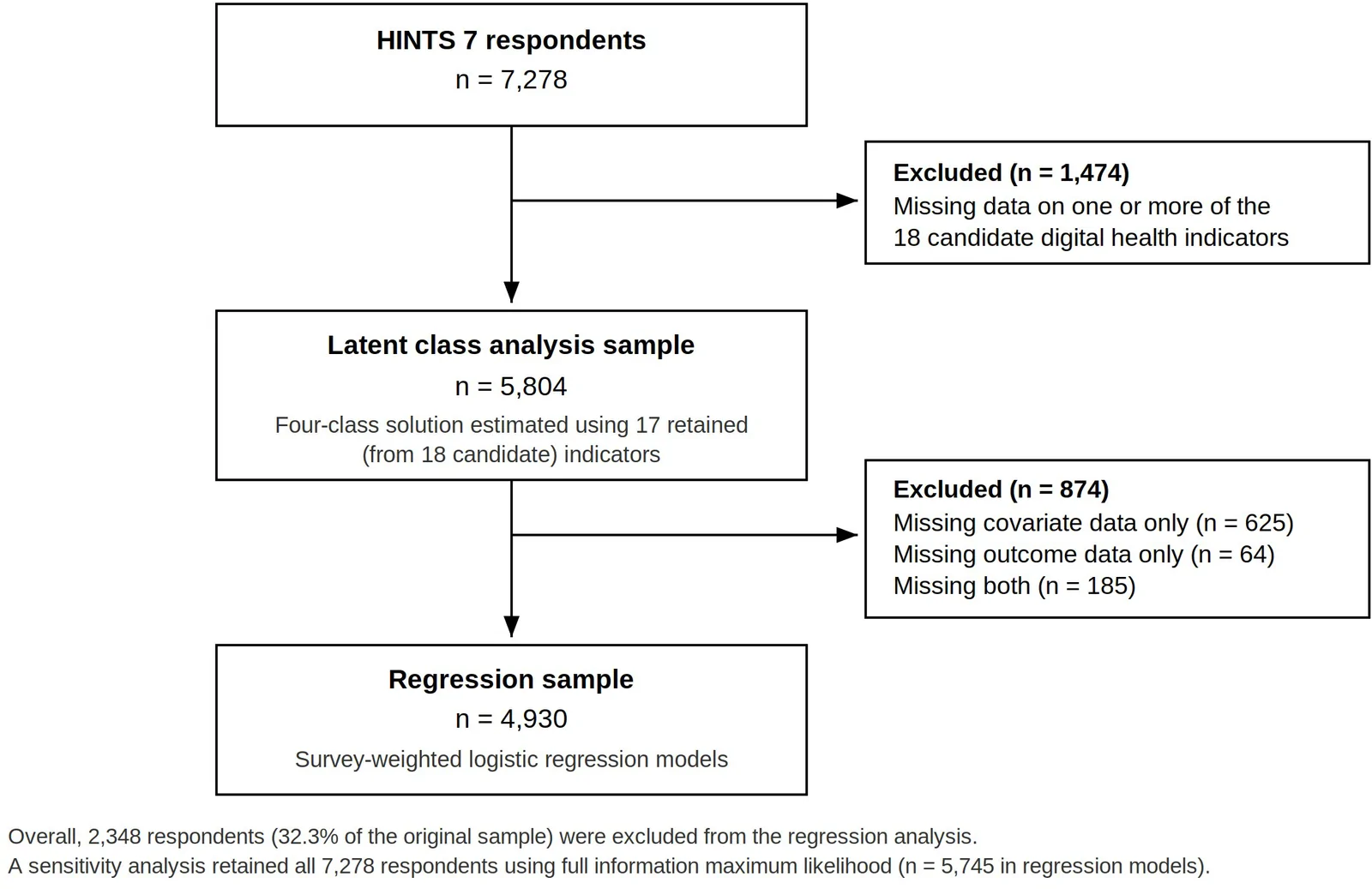
Flow diagram of analytic sample selection.

### 2.2 Measures

#### 2.2.1 Digital health behavior indicators

Candidate indicators were identified from HINTS 7 items capturing respondents’ use of digital tools or services for health-related activities, together with platform-use indicators that provided context for health-specific wearable and social media behaviors. Items limited to general internet access, attitudes, perceived skills, or evaluations of care were not considered. The general social media-use indicator was retained as a contextual indicator and was not interpreted as evidence of health-related engagement by itself. This yielded 18 candidate binary indicators, which were organized into five domains for presentation and interpretation: wearable device use; mobile health applications and data sharing; online health information and patient portal engagement; social media health engagement; and telehealth use. Example items, each with a 12-month reference period, include “have you used an electronic wearable device to monitor or track your health or activity?” (wearable device use), “have you used a health or wellness app on your tablet or smartphone?” (mobile health applications and data sharing), “have you used the Internet to look for health or medical information?” and “how many times did you access your online medical record or patient portal?” (online health information and patient portal engagement), “how often did you share general health-related information on social media?” (social media health engagement), and “did you receive care from a doctor or health professional using telehealth?” (telehealth use). Online health information seeking and patient portal engagement were placed in the same domain because both involve internet-mediated engagement with health information, clinical information, or care-related services, although they represent distinct functions. Each indicator was coded 1 if the respondent reported the behavior and 0 otherwise. Nonapplicable responses generated by questionnaire skip logic were coded as structural zeros rather than treated as missing, reflecting the interpretation that respondents not administered a follow-up item had not engaged in the corresponding behavior; refusals, “don’t know” responses, and commission-error codes were treated as missing. Inspection of phi-coefficient correlations among the candidates showed that the portal-specific item for viewing test results was strongly correlated with any patient-portal access (φ ≈ 0.86), raising concern about strong local dependence in the LCA model (Collins and Lanza, 2010). Because a separate and more general indicator for retrieving medical test results via the internet was already included, the portal-specific test-results item was excluded. The final LCA indicator set therefore included 17 retained indicators, which are listed with their HINTS source items, coding, and weighted endorsement rates in **Supplementary Table 1**. All 17 indicators were entered individually into the latent class model, and the five domains were used for organization and interpretation rather than as validated subscales.

#### 2.2.2 Physical activity outcomes

Adherence to the Physical Activity Guidelines for Americans (U.S. Department of Health and Human Services, 2018) was operationalized using two HINTS-derived behavioral measures. Aerobic guideline adherence was defined as at least 150 minutes per week of moderate-intensity exercise. Because HINTS 7 did not separately quantify vigorous-intensity minutes, aerobic adherence was operationalized as a conservative proxy based on moderate-intensity activity only. Muscle-strengthening adherence was defined as muscle-strengthening activity on at least 2 days per week (U.S. Department of Health and Human Services, 2018). The primary outcome was concurrent adherence to both aerobic and muscle-strengthening guidelines; aerobic adherence and muscle-strengthening adherence were examined separately as secondary outcomes.

#### 2.2.3 Covariates

Covariates were specified *a priori* to account for sociodemographic, socioeconomic, and health-related characteristics associated with both digital health engagement and physical activity in prior population-based research. Selection was informed by the covariate sets used in prior HINTS-based analyses of digital health behavior and physical activity (Lee et al., 2025; Xie et al., 2020) rather than by statistical significance in those studies or in the present sample. All covariates were entered simultaneously, and no variable selection procedure was applied. Demographic characteristics included age group (18–34, 35–49, 50–64, 65–74, and ≥75 years), sex assigned at birth (female, male, and unknown), education (less than high school, high school graduate, some college, and college graduate or higher), and race and ethnicity (Hispanic, non-Hispanic White, non-Hispanic Black, non-Hispanic American Indian/Alaska Native, non-Hispanic Asian, non-Hispanic Native Hawaiian/Pacific Islander, and non-Hispanic multiple race). Socioeconomic and health characteristics included household income (<$20,000, $20,000–$34,999, $35,000–$49,999, $50,000–$74,999, $75,000–$99,999, and ≥$100,000), body mass index (continuous, kg/m²), psychological distress (Patient Health Questionnaire-4 [PHQ-4] total score; Kroenke et al., 2009), smoking status (never, former, and current), and marital status (married, living with partner, divorced, widowed, separated, and single).

### 2.3 Statistical analysis

The LCA sample was defined at the candidate-indicator screening stage as respondents with complete data on all 18 candidate indicators (n = 5,804). This sample definition was kept after the redundant portal item was removed, because redefining the sample on the 17 retained indicators would have added only 5 respondents. LCA was then conducted in Mplus 8.11 (Muthén and Muthén, 1998–2017) on the 17 retained binary indicators in this sample. The influence of this complete-case approach was evaluated in a sensitivity analysis described below. Candidate models with 2 through 8 classes were estimated using maximum likelihood with robust standard errors, incorporating the HINTS final person-level sampling weight to improve population representativeness of class estimation; replicate weights were reserved for the regression stage. To reduce the risk of local maxima, each model was estimated using multiple random starting values. The best log-likelihood was replicated across starts for all candidate models, indicating stable model solutions. Class enumeration was evaluated using both statistical and substantive criteria (Nylund-Gibson and Choi, 2018; Weller et al., 2020). Statistical criteria included the Akaike Information Criterion (AIC), the Bayesian Information Criterion (BIC), the sample-size adjusted BIC (aBIC), the Lo-Mendell-Rubin adjusted likelihood ratio test (LMR-aLRT) (Lo et al., 2001) comparing each k-class solution to the (k−1)-class solution, classification entropy, and the smallest-class proportion. The substantive criterion was the interpretability of the resulting profiles. The bootstrap likelihood ratio test was not used because it is not available in Mplus when sampling weights are applied. After the final solution was selected, class labels were assigned *post hoc* based on item-response probability patterns across the five behavioral domains.

Associations between latent class membership and physical activity guideline adherence were examined using survey-weighted logistic regression in R version 4.4.0 (R Core Team, 2024) with the survey package (Lumley, 2024). The complex sample design was specified using the 50 jackknife replicate weights with a jackknife adjustment factor of 0.98 and delete-one jackknife (JK1) variance estimation. For each of the three binary outcomes, namely concurrent adherence to both guidelines, aerobic adherence, and muscle-strengthening adherence, we fitted a crude model with latent class membership as the sole predictor and an adjusted model that additionally included all covariates listed above. The Limited Engagement class served as the reference category, and overall class effects were tested with Wald F-tests. Statistical significance was set at α = .05 with 95% confidence intervals. To compare the two intermediate profiles directly, *post hoc* adjusted contrasts were estimated by reparameterizing the fully adjusted models with the Portal-Centered Engagement class as the reference. These exploratory contrasts were not adjusted for multiple comparisons.

To evaluate the potential influence of the complete-case approach, we conducted a sensitivity analysis in which the four-class model was re-estimated on the full sample (N = 7,278) using full information maximum likelihood for missing indicator data under a missing at random assumption. We compared class proportions, item-response probabilities, and classification quality with those of the primary model, and cross-tabulated modal class assignment for participants common to both models. Survey-weighted logistic regression models were then re-estimated using class assignment derived from the sensitivity model. Information criteria were not compared across the two models because they were estimated on different numbers of observations.

## 3 Results

### 3.1 Analytic sample and outcome prevalence

Of the 7,278 HINTS 7 respondents, 5,804 (79.7%) had complete data on all 18 candidate digital health behavior indicators screened for inclusion and constituted the LCA sample; 1,474 respondents (20.3%) were excluded because of missing data on at least one candidate indicator. Among the 17 indicators retained in the final LCA model, item-level missingness ranged from 0.8% to 10.5%. Missingness was greatest for willingness to share wearable device data (10.5%) and frequency of wearable tracking (10.4%), which together accounted for 40.5% of all missing indicator responses. Of the 5,804 LCA respondents, 4,930 (84.9%) also had complete data on the physical activity outcomes and all covariates and constituted the regression sample. Of the 874 respondents excluded at this stage, 625 were missing covariate data only, 64 were missing physical activity outcome data only, and 185 were missing both. Overall, 2,348 respondents (32.3% of the original sample) were excluded from the regression analysis. In the regression sample, 41.8% of respondents met the aerobic guideline, 41.3% met the muscle-strengthening guideline, and 25.1% met both guidelines concurrently.

Participants excluded from the LCA sample differed substantially from those included. Excluded participants were markedly older, with 52.7% aged 65 years or older compared with 17.7% of included participants (p <.001), and had lower educational attainment (15.3% with less than a high school education versus 4.9%; p <.001) and lower household income (24.5% with household income below $20,000 versus 13.7%; p <.001). Differences were also observed in marital status (p <.001) and race and ethnicity (p = .020). Excluded participants reported slightly lower psychological distress (mean PHQ-4 score 1.99 versus 2.42; p = .006), whereas sex, smoking status, and body mass index did not differ significantly (all p >.27). Within the LCA sample, respondents excluded from the regression analysis were more likely to belong to the Limited Engagement class (33.6% versus 19.4%; p <.001). Full comparisons are presented in **Supplementary Table 4**.

### 3.2 Latent class enumeration and selection

Fit statistics for the 2- through 8-class candidate models are presented in **Table 1**. The BIC decreased monotonically from 101,809 at k = 2 to 90,172 at k = 8. The LMR-aLRT supported sequential class additions up to k = 4 (p = .014) and was non-significant for all higher-order solutions (k = 5: p = .359; k = 6: p = .314; k = 7: p = .517; k = 8: p = .446), indicating that additional classes beyond four did not provide statistically significant improvement. Classification entropy remained high for the 4-class solution (0.911) and decreased thereafter. The smallest class proportion was 11.8% at k = 4 but fell below 10% at k ≥ 6, raising concerns about stability and interpretability for higher-order models.

**Table 1:** Model fit statistics for latent class analysis models with 2 to 8 classes (N = 5,804).

Classes (k)	Free parameters	Log-likelihood	AIC	BIC	aBIC	Entropy	Smallest class (%)	LMR-aLRT p
2	35	−50,752.86	101,575.7	101,809.0	101,697.8	0.985	43.3	—
3	53	−47,521.62	95,149.2	95,502.6	95,334.1	0.928	23.2	<.001
**4**	**71**	**−46,262.94**	**92,667.9**	**93,141.2**	**92,915.6**	**0.911**	**11.8**	**.014**
5	89	−45,681.45	91,540.9	92,134.2	91,851.4	0.904	11.0	.359
6	107	−45,200.66	90,615.3	91,328.6	90,988.6	0.887	7.6	.314
7	125	−44,755.63	89,761.3	90,594.6	90,197.3	0.860	7.6	.517
8	143	−44,466.53	89,219.1	90,172.3	89,717.9	0.865	6.5	.446

AIC, Akaike Information Criterion; BIC, Bayesian Information Criterion; aBIC, sample-size adjusted BIC; LMR-aLRT, Lo-Mendell-Rubin adjusted likelihood ratio test, comparing the k-class solution to the (k − 1)-class solution. Bold values indicate the 4-class solution retained as the final model on the basis of statistical fit indices, classification entropy, smallest-class proportion, and substantive interpretability.

Considered together with substantive interpretability, the 4-class solution yielded conceptually distinct and parsimonious profiles, whereas additional classes produced subdivisions of existing groups without introducing qualitatively new patterns. Accordingly, the 4-class solution was retained as the final model. Classification quality was high, with average posterior probabilities for the assigned class ranging from 0.92 to 0.96.

### 3.3 Description of the four latent classes

Model-estimated class proportions in the LCA sample were 32.0% for Broad Multidomain Engagement, 11.8% for Wearable-Centered Engagement, 34.3% for Portal-Centered Engagement, and 21.9% for Limited Engagement. **Supplementary Table 2** presents the estimated item-response probabilities of the 17 digital health behavior indicators across the four classes and observed weighted endorsement rates by class are reported in **Supplementary Table 3** and closely correspond to the model-estimated probabilities.

The classes were distinguished primarily by the joint patterning of wearable and patient portal behaviors. The Broad Multidomain Engagement class was characterized by high endorsement across all five domains, with probabilities exceeding .86 for wearable device use, health application use, and every patient portal indicator; it was the only class in which wearable-based self-monitoring and clinically oriented portal use were simultaneously common. The Wearable-Centered Engagement class showed high endorsement of the wearable indicators (.62 to 1.00) and willingness to share wearable data (.69), together with moderate health application use (.62), but substantially lower engagement with patient portal and provider communication behaviors (.24 to .49) and low telehealth use (.15). The Portal-Centered Engagement class showed the reverse pattern, with high endorsement of patient portal access (.90), provider messaging (.89), viewing test results online (.95), and online appointment scheduling (.82), but minimal wearable device use or tracking (≤.09). The Limited Engagement class showed low endorsement across most indicators, particularly clinically oriented behaviors (.10 to .27), although general social media use (.79), online health information seeking (.61), and health-related video viewing (.49) remained relatively common. This class served as the reference category in subsequent regression analyses.

### 3.4 Sample characteristics by latent class

Weighted demographic and health characteristics of the regression sample, overall and by latent class, are presented in **Table 2**. The four classes differed significantly across most demographic and health characteristics, including age, sex, education, household income, smoking status, marital status, race and ethnicity, and body mass index, whereas psychological distress did not differ significantly across classes. In general, the Broad Multidomain Engagement class had the highest proportion of college graduates and adults with household income of $100,000 or more, whereas the Limited Engagement class had lower educational attainment, lower household income, and a higher proportion of older adults.

**Table 2:** Weighted sample characteristics of the regression sample, overall and by latent class (N = 4,930).

Characteristic	Overall (n = 4,930)	Limited engagement (n = 1,024)	Wearable-centered (n = 481)	Portal-centered (n = 1,801)	Broad multidomain (n = 1,624)	p
Age group (%)						<.001
18–34	28.6	31.4	41.6	24.4	27.1	
35–49	28.8	21.0	25.8	28.1	35.2	
50–64	26.4	25.5	23.8	27.4	26.6	
65–74	10.4	11.8	6.2	13.7	7.6	
≥75	5.8	10.2	2.6	6.5	3.6	
Sex (%)						<.001
Female	48.5	34.9	39.7	51.0	56.8	
Male	51.0	64.8	59.6	48.5	42.9	
Unknown	0.4	0.4	0.7	0.5	0.3	
Education (%)						<.001
Less than high school	4.8	12.9	3.3	4.2	1.1	
High school graduate	19.6	27.4	19.7	20.9	13.5	
Some college	39.1	40.2	38.9	39.5	38.0	
College graduate or higher	36.6	19.6	38.1	35.4	47.3	
Race and ethnicity (%)						.024
Hispanic	16.7	22.2	22.1	13.6	14.9	
Non-Hispanic White	61.5	52.3	61.1	64.9	63.5	
Non-Hispanic Black	10.3	10.5	8.5	9.4	11.7	
NH American Indian/Alaska Native	0.5	1.5	0.0	0.5	0.2	
Non-Hispanic Asian	6.2	7.9	3.8	6.9	5.4	
NH Native Hawaiian/Pacific Islander	0.3	0.0	0.0	0.5	0.2	
Non-Hispanic Multiple	4.5	5.6	4.5	4.3	4.1	
Household income (%)						<.001
<$20,000	13.4	25.7	11.4	11.7	8.6	
$20,000–34,999	10.8	17.0	12.3	11.5	5.9	
$35,000–49,999	11.4	11.8	10.3	14.3	8.6	
$50,000–74,999	15.4	14.6	14.1	17.6	14.0	
$75,000–99,999	13.0	10.1	14.7	13.9	13.2	
≥$100,000	36.1	20.8	37.2	31.0	49.8	
Smoking status (%)						<.001
Never	65.0	65.9	73.6	57.8	69.3	
Former	23.0	20.1	13.3	27.5	23.2	
Current	12.0	13.9	13.1	14.7	7.5	
Marital status (%)						<.001
Married	51.3	41.8	43.8	48.3	62.5	
Living with partner	6.0	7.6	7.3	6.0	4.6	
Divorced	5.8	7.0	4.2	6.9	4.6	
Widowed	3.2	7.0	1.8	2.7	2.0	
Separated	1.4	1.1	0.6	1.9	1.5	
Single	32.3	35.4	42.3	34.2	24.9	
Body mass index (kg/m²), mean (SD)	28.8 (7.1)	28.1 (6.2)	27.8 (5.9)	28.9 (7.5)	29.5 (7.4)	.004
PHQ-4 score, mean (SD)	2.5 (3.1)	2.4 (3.3)	2.2 (2.9)	2.7 (3.4)	2.4 (2.9)	.280
Physical Activity Guideline adherence (%)						<.001
Aerobic	41.8	40.2	49.1	34.5	48.0	
Muscle-strengthening	41.3	32.5	43.4	37.8	49.5	
Both met	25.1	18.2	31.4	19.9	32.7	

Values are weighted percentages for categorical variables and weighted means (SD) for continuous variables. Unweighted Ns are shown in column headers. p-values are from Rao-Scott chi-square tests (categorical) and survey-weighted F-tests (continuous), with the complex sample design accounted for via 50 jackknife replicate weights. PHQ-4 = Patient Health Questionnaire-4.

Adherence to the physical activity guidelines also differed significantly across classes (all p <.001). The proportion meeting both guidelines was highest in the Broad Multidomain Engagement (32.7%) and Wearable-Centered Engagement (31.4%) classes and considerably lower in the Portal-Centered Engagement (19.9%) and Limited Engagement (18.2%) classes. For muscle-strengthening adherence, a graded pattern was observed, ranging from 32.5% in the Limited Engagement class to 49.5% in the Broad Multidomain Engagement class. For aerobic adherence, however, the Portal-Centered Engagement class showed the lowest proportion of all four classes (34.5%), below that of the Limited Engagement reference class (40.2%).

### 3.5 Latent class membership and physical activity guideline adherence

Crude and adjusted odds ratios from survey-weighted logistic regression models for adherence to the Physical Activity Guidelines are presented in **Table 3**. For the primary outcome of meeting both the aerobic and muscle-strengthening guidelines, latent class membership was significantly associated with adherence in both crude (Wald F(3, 46) = 15.25, p <.001) and adjusted (Wald F(3, 17) = 13.25, p <.001) models. Compared with the Limited Engagement reference class, the Broad Multidomain Engagement class had the strongest association (adjusted odds ratio [aOR] = 2.79, 95% confidence interval [CI]: 1.81–4.30, p <.001), followed by the Wearable-Centered Engagement class (aOR = 2.13, 95% CI: 1.42–3.19, p = .001). The Portal-Centered Engagement class showed a smaller association that did not reach statistical significance (aOR = 1.36, 95% CI: 0.97–1.89, p = .069).

**Table 3:** Crude and adjusted odds ratios for physical activity guideline adherence by latent class.

Class (vs. limited engagement)	Crude OR (95% CI)	p	Adjusted OR (95% CI)	p
Both guidelines met (primary outcome)				
Wearable-Centered	2.06 (1.33–3.19)	.002	2.13 (1.42–3.19)	.001
Portal-Centered	1.12 (0.80–1.56)	.512	1.36 (0.97–1.89)	.069
Broad Multidomain	2.18 (1.49–3.20)	<.001	2.79 (1.81–4.30)	<.001
Aerobic adherence				
Wearable-Centered	1.44 (0.99–2.08)	.056	1.38 (0.95–2.01)	.083
Portal-Centered	0.79 (0.59–1.04)	.090	0.87 (0.65–1.17)	.338
Broad Multidomain	1.38 (1.02–1.85)	.035	1.64 (1.17–2.29)	.007
Muscle-strengthening adherence				
Wearable-Centered	1.59 (1.07–2.37)	.023	1.64 (1.06–2.53)	.029
Portal-Centered	1.26 (0.99–1.61)	.061	1.58 (1.15–2.17)	.007
Broad Multidomain	2.03 (1.57–2.63)	<.001	2.58 (1.85–3.60)	<.001

OR, odds ratio; CI, confidence interval. Adjusted models include age group, sex, education, race and ethnicity, household income, body mass index, PHQ-4 score, smoking status, and marital status as covariates. All models use survey-weighted logistic regression with 50 jackknife replicate weights (JK1; jackknife adjustment factor = 0.98; 49 design degrees of freedom). Overall class effects are tested via Wald F-tests; denominator degrees of freedom for adjusted models reflect the additional estimated covariate parameters. Overall class effects were statistically significant for all three outcomes in both crude and adjusted models (all p <.001).

Patterns of association differed when aerobic and muscle-strengthening adherence were examined separately. For aerobic adherence, overall class membership was significantly associated with the outcome in both crude (Wald F(3, 46) = 11.04, p <.001) and adjusted (Wald F(3, 17) = 9.72, p <.001) models, but only the Broad Multidomain Engagement class differed significantly from the Limited Engagement reference (aOR = 1.64, 95% CI: 1.17–2.29, p = .007). The Wearable-Centered Engagement class had higher odds, but the association did not reach statistical significance (aOR = 1.38, 95% CI: 0.95–2.01, p = .083), and the Portal-Centered Engagement class did not differ from the reference (aOR = 0.87, 95% CI: 0.65–1.17, p = .338).

For muscle-strengthening adherence, overall class membership was again significant in both crude (Wald F(3, 46) = 12.30, p <.001) and adjusted (Wald F(3, 17) = 12.69, p <.001) models, and all three non-reference classes differed significantly from the Limited Engagement reference: Broad Multidomain Engagement (aOR = 2.58, 95% CI: 1.85–3.60, p <.001), Wearable-Centered Engagement (aOR = 1.64, 95% CI: 1.06–2.53, p = .029), and Portal-Centered Engagement (aOR = 1.58, 95% CI: 1.15–2.17, p = .007). Overall, associations differed across latent classes and physical activity outcomes: only the most comprehensively engaged profile was significantly associated with aerobic guideline adherence, whereas all digitally engaged profiles were associated with higher odds of muscle-strengthening adherence.

In *post hoc* adjusted contrasts using the Portal-Centered Engagement class as the reference, the Wearable-Centered Engagement class had higher adjusted odds of meeting both guidelines (aOR = 1.57, 95% CI: 1.04–2.37, p = .034) and of meeting the aerobic guideline (aOR = 1.59, 95% CI: 1.06–2.38, p = .029). No evidence of a difference between the two classes was observed for muscle-strengthening adherence (aOR = 1.04, 95% CI: 0.71–1.51, p = .845). These contrasts were exploratory and were not adjusted for multiple comparisons.

In a sensitivity analysis using full information maximum likelihood to retain respondents with partially missing indicator data, all 7,278 respondents contributed to the latent class analysis and 5,745 to the regression analyses. The four-class structure was reproduced with comparable class proportions (Broad Multidomain Engagement 29.2%, Wearable-Centered Engagement 11.0%, Portal-Centered Engagement 36.7%, Limited Engagement 23.1%) and classification quality (entropy = .914 versus .911), and modal class assignment agreed for 97.7% of the 5,804 respondents included in the primary analysis (κ = 0.97). Adjusted associations were generally similar in direction and magnitude, although two associations that did not reach statistical significance in the primary analysis were statistically significant in the larger sample: Portal-Centered Engagement for meeting both guidelines (aOR = 1.50, 95% CI: 1.13–2.00) and Wearable-Centered Engagement for aerobic adherence (aOR = 1.45, 95% CI: 1.02–2.08). Full comparisons are presented in **Supplementary Table 5**.

## 4 Discussion

### 4.1 Principal findings and interpretation

Using nationally representative data from HINTS 7, we identified four qualitatively distinct profiles of digital health engagement among U.S. adults: Broad Multidomain Engagement, Wearable-Centered Engagement, Portal-Centered Engagement, and Limited Engagement. These profiles were derived from 17 indicators spanning five behavioral domains. Profile membership was associated with adherence to the Physical Activity Guidelines for Americans, but the strength and consistency of these associations varied by profile and by physical activity outcome. Relative to the Limited Engagement profile, the Broad Multidomain Engagement and Wearable-Centered Engagement profiles were most strongly associated with meeting both guidelines concurrently. In the primary complete-case models, aerobic guideline adherence was significantly higher only in the Broad Multidomain Engagement profile, whereas all three digitally engaged profiles had higher odds of muscle-strengthening adherence. These findings suggest that configurations of digital health behaviors may better characterize heterogeneity in physical activity adherence among U.S. adults than single-behavior measures alone.

Prior HINTS-based studies have primarily examined individual digital health behaviors, especially wearable device or mobile health app use, as isolated correlates of physical activity, consistently reporting positive associations. For example, prior studies reported adjusted odds ratios of 1.53 and 1.84 for meeting the aerobic guideline among wearable users (Lee et al., 2025; Xie et al., 2020). These findings are consistent with systematic review evidence suggesting that consumer-based wearable activity trackers can support physical activity participation through self-monitoring and feedback when incorporated into behavioral interventions (Brickwood et al., 2019). Latent class analysis has been applied to HINTS for other purposes, including eHealth practices among cancer survivors (Lin et al., 2020) and trust typologies in health information (Stimpson et al., 2025). The most directly comparable study classified adults by digital technology access and use but treated physical activity only as a correlate of class membership rather than as the primary outcome (Hegeman et al., 2024). The present study extends this work by linking multi-domain digital health profiles directly to physical activity guideline adherence.

The present findings suggest that digital health engagement is organized into distinct behavioral configurations, and the separation between the Wearable-Centered Engagement and Portal-Centered Engagement profiles is particularly informative. Both profiles reflected active digital health engagement, but their behavioral content and associations with physical activity adherence differed. The Broad Multidomain Engagement profile showed the most consistent associations across physical activity outcomes, whereas the Wearable-Centered Engagement profile was associated with concurrent and muscle-strengthening adherence but not aerobic adherence, and the Portal-Centered Engagement profile showed a more selective association with muscle-strengthening adherence alone. This pattern suggests that overall digital engagement may not be best understood as a single homogeneous construct, although the cross-sectional design precludes causal interpretation.

The Wearable-Centered Engagement profile may reflect a more direct connection between digital self-monitoring and physical activity behavior. Wearable activity trackers are designed to capture movement-related feedback, support goal setting, and make activity patterns visible to users, which may help explain why this profile was associated with concurrent and muscle-strengthening guideline adherence. However, this association should not be interpreted as evidence that wearable use itself causes guideline adherence. Wearable users may differ systematically from non-users, and surveillance research suggests that physical activity estimates based only on wearable users may overestimate population activity levels (Hyde et al., 2020). The Wearable-Centered Engagement profile may therefore represent both technology-enabled self-monitoring and a pre-existing orientation toward physical activity.

The Portal-Centered Engagement profile requires a different interpretation. Patient portals are not primarily designed for activity tracking, but they may reflect broader engagement with health management, clinical information, and patient-provider communication. Systematic review evidence suggests that patient portals can support access to health information, improve health status awareness, strengthen patient-provider interaction, and increase adherence to care, although their effects on health service utilization and efficiency remain mixed (Carini et al., 2021). In the present study, this broader health-management orientation may help explain why Portal-Centered Engagement membership was associated with muscle-strengthening adherence but not with aerobic or concurrent adherence after adjustment.

A notable finding of this study is that the pattern of statistically significant associations differed descriptively between the aerobic and muscle-strengthening outcomes. Whereas only the Broad Multidomain Engagement profile was significantly associated with aerobic guideline adherence, every digitally engaged profile was associated with higher odds of muscle-strengthening adherence. Differences between the class contrasts across the two outcomes were not formally tested. This outcome-specific pattern may reflect differences in how aerobic and muscle-strengthening activities are supported and monitored through digital health engagement. Muscle-strengthening activity may represent a more deliberate and planned form of health behavior, which could align not only with wearable-based self-monitoring but also with the broader health-management orientations reflected in portal and online health-information engagement. Aerobic guideline adherence, by contrast, may be achieved through more heterogeneous forms of activity, including both structured exercise and lifestyle-based movement, which may weaken its alignment with any single pattern of digital health engagement.

At the same time, the aerobic and muscle-strengthening findings should be interpreted in light of measurement differences between the two outcomes. Because HINTS 7 did not separately quantify vigorous-intensity activity, aerobic guideline adherence was operationalized as a conservative proxy based on moderate-intensity minutes alone. This proxy may misclassify adults who meet the aerobic guideline primarily through vigorous activity, and this measurement constraint may have attenuated or otherwise biased associations for the aerobic outcome. The observed outcome-specific pattern should therefore be interpreted cautiously, as it is consistent with a true behavioral difference, a measurement artifact, or a combination of both. Disentangling these explanations will require data sources that capture both moderate- and vigorous-intensity activity with comparable fidelity.

The sociodemographic composition of the four profiles provides additional context for the observed patterns of digital health behavior. Educational attainment and household income most clearly distinguished the Limited Engagement profile from the other three: 12.9% had less than a high school education and 25.7% reported household incomes below $20,000, compared with 1.1% to 4.2% and 8.6% to 11.7%, respectively, across the remaining profiles. The Broad Multidomain Engagement profile had the largest observed proportions of college graduates (47.3%), adults with household incomes of $100,000 or more (49.8%), and married adults (62.5%), together with the lowest prevalence of current smoking (7.5%). This socioeconomic gradient is consistent with prior HINTS evidence that older and less educated adults are less likely to belong to classes characterized by broad digital access and use (Hegeman et al., 2024), and that sociodemographic characteristics predict engagement across multiple digital health behaviors (Ratcliff et al., 2021).

The two intermediate profiles were similar in socioeconomic composition but differed primarily by age and sex. Adults aged 65 years or older were approximately half as prevalent in the two profiles involving wearable use (8.8% in Wearable-Centered Engagement and 11.2% in Broad Multidomain Engagement) as in the two profiles without substantial wearable involvement (22.0% in Limited Engagement and 20.2% in Portal-Centered Engagement), consistent with evidence that wearable adoption declines with age (Chandrasekaran et al., 2020). Sex, by contrast, tracked portal engagement: women comprised 34.9% and 39.7% of the Limited Engagement and Wearable-Centered Engagement profiles but 51.0% and 56.8% of the Portal-Centered Engagement and Broad Multidomain Engagement profiles. Because portal access and portal-based communication generally require an established connection with a health care organization, this pattern may partly reflect opportunities for engagement created through health care contact rather than technology preference alone. Although men were more common in the Wearable-Centered Engagement profile than in the portal-involving profiles, prior HINTS analyses treating wearable use as a single behavior reported higher wearable use among women (Lee et al., 2025). This contrast underscores that profiles defined by multiple co-occurring behaviors are not interchangeable with single-indicator measures of technology use.

These profiles should not be interpreted as a simple gradient from poorer to better health: the Broad Multidomain Engagement profile had the highest mean body mass index (29.5 kg/m²), and psychological distress did not differ significantly across profiles. The physical activity pattern also did not simply follow the socioeconomic gradient. Despite having higher educational attainment and household income than the Limited Engagement profile, the Portal-Centered Engagement profile had a lower weighted prevalence of aerobic guideline adherence (34.5% versus 40.2%) and a similar prevalence of concurrent adherence to both guidelines (19.9% versus 18.2%). The Wearable-Centered and Portal-Centered profiles had broadly comparable socioeconomic compositions. In *post hoc* adjusted contrasts, the Wearable-Centered profile had higher odds of aerobic and concurrent adherence, whereas no evidence of a difference was observed for muscle-strengthening adherence. Because differences between these contrast estimates across outcomes were not formally tested, and because the contrasts were not adjusted for multiple comparisons, this descriptive pattern should not be interpreted as demonstrating that the class contrast was stronger for one outcome than another. These patterns suggest that the configuration of digital health engagement may provide information beyond socioeconomic advantage alone. In the adjusted models reported above, the overall association between class membership and each physical activity outcome remained statistically significant after accounting for the measured sociodemographic and health characteristics. Nevertheless, residual confounding by unmeasured aspects of socioeconomic position, health care access, digital skills, and pre-existing activity orientation cannot be excluded.

### 4.2 Translational implications for adaptive digital health support

The present findings may have translational implications for adaptive digital health support, including future artificial intelligence (AI)-enabled intervention systems. Rather than tailoring physical activity support based only on whether an individual uses a single technology, such as a wearable device or mobile health app, future systems could use broader digital health engagement profiles as an initial personalization layer. For example, adults in a Wearable-Centered Engagement profile may benefit from wearable feedback, self-monitoring, and goal-setting modules, whereas Portal-Centered Engagement users may benefit from patient portal messaging, appointment-linked prompts, or clinician-integrated feedback. Broad Multidomain Engagement users may be more receptive to multi-channel strategies, while adults in the Limited Engagement profile may require hybrid approaches that combine digital tools with community-based or in-person support.

In future AI-enabled implementations, profile assignment would need to be updated and supplemented with ongoing user data rather than being treated as a fixed baseline classification. Future systems may integrate profile membership with ongoing behavioral and wearable-derived data to personalize physical activity support. The descriptive pattern across outcomes observed here raises the possibility that aerobic and muscle-strengthening support may require different adaptation strategies. For instance, a system might use wearable feedback to adjust aerobic goals while using portal- or app-based education, scheduling prompts, and progression tracking to support muscle-strengthening behavior.

These implications remain hypothesis-generating because the present study did not develop or test an AI-enabled intervention and did not include real-time wearable biomarkers or objective physiological outcomes. Future longitudinal and experimental studies are needed to determine whether profile-informed adaptive systems can improve physical activity adherence and physiological outcomes. Such studies should incorporate objective measures, such as device-measured activity intensity, heart rate variability, cardiorespiratory indicators, or direct measures of muscle strength, to evaluate whether adaptive personalization improves both behavior and physiological response.

Because the identified profiles were socially patterned, future profile-informed systems should also be designed with attention to equity. Adaptive interventions may otherwise work best for users who are already highly engaged with digital health technologies, potentially widening existing disparities in physical activity support. This concern is concrete in the present data: the Limited Engagement profile included the largest observed proportions of adults aged 75 years or older, adults with less than a high school education, and those with household incomes below $20,000, and had the lowest observed prevalence of meeting both guidelines. Future systems should therefore be evaluated not only for overall effectiveness but also for whether benefits are distributed equitably across users with different levels of digital health engagement.

### 4.3 Strengths and limitations

This study has several strengths, including the use of a large, nationally representative sample, incorporation of survey weights in class estimation and replicate weights in regression variance estimation, a multi-domain indicator set spanning the contemporary digital health ecosystem, a transparent latent class enumeration procedure evaluated against multiple statistical and substantive criteria, and a sensitivity analysis evaluating the influence of missing indicator data on the identified profiles and their associations.

Several limitations should be acknowledged. First, the cross-sectional design precludes causal or temporal inference; it is not possible to determine whether digital health engagement promotes physical activity, whether physically active adults are more inclined to adopt digital health tools, or whether both are driven by shared underlying characteristics. Second, all measures were self-reported and subject to recall and social desirability biases; physical activity in particular is known to be over-reported in self-report instruments. Third, as discussed above, the aerobic outcome is a conservative proxy based on moderate-intensity activity only, because HINTS 7 did not separately quantify vigorous-intensity minutes; this measurement constraint may have attenuated associations for the aerobic outcome.

Fourth, latent class membership was assigned using each respondent’s most-likely class and was treated as observed in the regression models. This widely used approach does not account for classification uncertainty in subsequent regression models, and approaches that account for this uncertainty, such as the Bolck-Croon-Hagenaars (BCH) method or three-step maximum-likelihood methods, may yield slightly different estimates.

Fifth, participants with missing data on any candidate digital health indicator were excluded before the latent class analysis, and the regression sample further excluded respondents with missing physical activity outcomes or covariates, so that 32.3% of the original sample was excluded from the regression analysis. We acknowledge that latent class models can retain participants with partially observed indicator data using full information maximum likelihood, as applied in related work (Hegeman et al., 2024). Excluded participants differed from included participants across several characteristics, being markedly older and having lower educational attainment and household income, and respondents excluded from the regression analysis were disproportionately assigned to the Limited Engagement class. The analytic sample may therefore underrepresent older and socioeconomically disadvantaged adults, and both the estimated class distribution and the observed associations should be interpreted with this in mind. In a sensitivity analysis retaining all respondents through full information maximum likelihood, the four-class structure and the direction and magnitude of the associations were reproduced, although two associations that did not reach statistical significance in the primary analysis were statistically significant in the larger sample. Selection bias therefore cannot be excluded, particularly with respect to generalizability to adults with lower digital health engagement. The sensitivity analysis relies on a missing at random assumption that cannot be verified, and it addressed missing indicator data only; respondents with missing physical activity outcomes or covariates remained excluded from all regression models.

Sixth, because the adjusted survey-weighted models included multiple multi-category covariates, the denominator degrees of freedom for adjusted Wald tests were limited, which may have reduced the precision of some adjusted estimates. Seventh, the contrasts between the Wearable-Centered Engagement and Portal-Centered Engagement profiles were *post hoc* and exploratory and were not adjusted for multiple comparisons; they should be regarded as hypothesis-generating. Although sampling weights were used to improve population representativeness, residual nonresponse bias may remain given the overall survey response rate.

Finally, the latent class labels, such as Wearable-Centered Engagement and Limited Engagement, are descriptive summaries of probabilistic response patterns assigned *post hoc*; they should be understood as interpretive shorthand rather than as fixed or mutually exclusive behavioral types.

### 4.4 Conclusions

In a nationally representative sample of U.S. adults, digital health engagement was organized into four distinct multi-domain profiles, and these profiles were differentially associated with physical activity guideline adherence. Associations were most consistent for muscle-strengthening activity and for profiles involving wearable-based self-monitoring or broad multidomain engagement, whereas in the primary complete-case models, aerobic guideline adherence was associated only with the most comprehensively engaged profile. These findings suggest that combinations of digital health behaviors may provide an informative unit for understanding digital correlates of physical activity and for designing future adaptive digital health support.

From a translational perspective, these profiles may provide an initial foundation for profile-guided personalization, including future AI-enabled support systems that combine digital engagement patterns with objective activity and physiological data. Longitudinal and experimental research is needed to determine whether such systems can dynamically adjust physical activity recommendations, improve adherence, and support related health outcomes.

## Data Availability

Publicly available datasets were analyzed in this study. This data can be found here: National Cancer Institute Health Information National Trends Survey (HINTS 7, 2024), publicly available at https://hints.cancer.gov/data/download-data.aspx.
