# Supplementary material for: Digital health engagement profiles and physical activity guideline adherence among adults in the United States: a latent class analysis
Source: Front Physiol. 2026 Sep 16;17:1901594. doi: 10.3389/fphys.2026.1901594 (PMC13624967; doi:10.3389/fphys.2026.1901594)
Supplement: Supplementary file 1 [file SupplementaryFile1.docx]

**Supplementary Material**

**Supplementary Table S1.** *Digital health behavior indicators: HINTS 7 source items, coding, and weighted endorsement rates in the latent class analysis sample (N = 5,804)*

| **Domain** | **Indicator** | **HINTS 7 source item** | **Coding** | **Weighted % endorsing** |
| --- | --- | --- | --- | --- |
| **Wearable device use** | Owns/uses smartwatch or wearable device | B6d; UseDevice_SmWatch — used a smartwatch or wearable device in the past 12 months | 1 = yes; 0 = no | 42.3 |
|  | Uses wearable to track health or activity | B8; WearableDevTrackHealth2 — used a wearable device to monitor or track health or activity | 1 = yes; 0 = no / no device | 44.5 |
|  | Frequent wearable tracking | B9; FreqWearDevTrackHealth2 — frequency of wearable health tracking in the past month | 1 = every/almost every day; 0 = less frequent or not tracking† | 31.7 |
| **Mobile health apps and data sharing** | Used a health or wellness app | B7; UsedHealthWellnessApps2 — used a health or wellness app | 1 = yes; 0 = no | 60.7 |
|  | Shared device/app data with provider | B11; SharedHealthDeviceInfo2 — shared health information from a monitoring device or smartphone with a health professional | 1 = yes; 0 = no | 19.3 |
|  | Willing to share wearable data with provider | B10; WillingShareData_HCP2 — willing to share wearable health data with a health care provider | 1 = yes; 0 = no† | 32.7 |
| **Online health information and patient portal** | Sought health information online | B3a; Electronic2_HealthInfo — used the internet to look for health or medical information | 1 = yes; 0 = no† | 86.4 |
|  | Messaged provider online | B3b; Electronic2_MessageDoc — used the internet to send a message to a doctor or provider | 1 = yes; 0 = no† | 67.4 |
|  | Viewed test results online | B3c; Electronic2_TestResults — used the internet to view medical test results | 1 = yes; 0 = no† | 74.6 |
|  | Scheduled appointment online | B3d; Electronic2_MadeAppts — used the internet to make an appointment with a provider | 1 = yes; 0 = no† | 68.9 |
|  | Accessed patient portal | E3; AccessOnlineRecord3 — accessed an online medical record or patient portal at least once | 1 = yes; 0 = no | 73.1 |
|  | Viewed clinical notes via portal | E5b; RecordsOnline2_ViewNotes — used the patient portal to view clinical notes | 1 = yes; 0 = no† | 58.9 |
| **Social media health engagement** | Visited social media (any) | B12a; SocMed_Visited — visited a social media site at least sometimes | 1 = at least rarely; 0 = never | 91.0 |
|  | Shared health information on social media | B12b/B12c; SocMed_SharedPers / SocMed_SharedGen — shared personal or general health information on social media | 1 = at least rarely (either); 0 = never (both) | 34.8 |
|  | Interacted with similar-condition users | B12d; SocMed_Interacted — interacted with others having similar health conditions on social media | 1 = at least rarely; 0 = never | 28.0 |
|  | Watched health-related video | B12e; SocMed_WatchedVid — watched a health-related video on social media | 1 = at least rarely; 0 = never | 71.1 |
| **Telehealth** | Received telehealth care | D1; ReceiveTelehealthCare — received telehealth care in the past 12 months | 1 = yes; 0 = no | 36.1 |

*Note. Item numbers refer to the HINTS 7 questionnaire. Weighted endorsement is the population-weighted proportion endorsing the behavior (response = 1) in the latent class analysis sample (n = 5,804), estimated with the HINTS final person-level sampling weight. † Structural zeros: respondents not administered the item because of questionnaire skip logic (e.g., no wearable tracking for B9 and B10; internet non-use for B3a–B3d; no patient-portal access for E5b) were coded 0 (not engaged) rather than treated as missing. From an initial set of 18 candidate indicators, the portal-specific test-results item (E5a; RecordsOnline2_ViewResults) was excluded because of high collinearity with patient-portal access (φ ≈ 0.86), yielding the 17 indicators above.*

**Supplementary Table S2.** *Estimated item-response probabilities of the 17 digital health behavior indicators by latent class (four-class solution)*

| **Domain** | **Indicator** | **Class 1**  **Broad Multidomain (32.0%)** | **Class 2**  **Wearable-Centered (11.8%)** | **Class 3**  **Portal-Centered**  **(34.3%)** | **Class 4**  **Limited Engagement**  **(21.9%)** |
| --- | --- | --- | --- | --- | --- |
| **Wearable device use** | Owns/uses smartwatch or wearable device | **.875** | **.803** | .091 | .077 |
|  | Uses wearable to track health or activity | **1.000** | **1.000** | .014 | .011 |
|  | Frequent wearable tracking | **.764** | **.616** | .000 | .000 |
| **Mobile health apps and data sharing** | Used a health or wellness app | **.927** | **.620** | **.562** | .200 |
|  | Shared device/app data with provider | .269 | .120 | .214 | .086 |
|  | Willing to share wearable data with provider | **.767** | **.690** | .000 | .000 |
| **Online health information and patient portal** | Sought health information online | **.970** | **.781** | **.954** | **.614** |
|  | Messaged provider online | **.914** | .296 | **.887** | .194 |
|  | Viewed test results online | **.972** | .448 | **.946** | .265 |
|  | Scheduled appointment online | **.910** | .488 | **.817** | .274 |
|  | Accessed patient portal | **.969** | .460 | **.902** | .260 |
|  | Viewed clinical notes via portal | **.866** | .235 | **.765** | .098 |
| **Social media health engagement** | Visited social media (any) | **.969** | **.924** | **.926** | **.788** |
|  | Shared health information on social media | .494 | .212 | .360 | .189 |
|  | Interacted with similar-condition users | .424 | .138 | .301 | .114 |
|  | Watched health-related video | **.829** | **.706** | **.743** | .491 |
| **Telehealth** | Received telehealth care | **.500** | .153 | .444 | .140 |

*Note. Values are model-estimated probabilities of endorsing each indicator (response = 1) conditional on latent class membership, from the final four-class latent class analysis (Mplus 8.11, MLR estimator, sampling-weighted; N = 5,804). Column headers report the model-estimated class proportions. Probabilities are reported without a leading zero because they cannot exceed 1; shaded, bold values denote probabilities ≥ .50, shown to aid readability. Class labels were assigned post hoc from the relative patterning of item-response probabilities across the five behavioral domains.*

**Supplementary Table S3.** *Observed weighted endorsement rates of the 17 digital health behavior indicators, overall and by latent class (n = 5,804)*

| **Domain** | **Indicator** | **Overall** | **Class 1**  **Broad Multidomain (n = 1,821)** | **Class 2**  **Wearable-Centered (n = 591)** | **Class 3**  **Portal-Centered (n = 2,074)** | **Class 4**  **Limited Engagement (n = 1,318)** |
| --- | --- | --- | --- | --- | --- | --- |
| **Wearable device use** | Owns/uses smartwatch or wearable device | 42.3 | **87.7** | **80.6** | 8.7 | 7.7 |
|  | Uses wearable to track health or activity | 44.5 | **100.0** | **100.0** | 1.3 | 1.2 |
|  | Frequent wearable tracking | 31.7 | **75.9** | **62.1** | 0.0 | 0.0 |
| **Mobile health apps and data sharing** | Used a health or wellness app | 60.7 | **92.3** | **61.7** | **56.3** | 19.1 |
|  | Shared device/app data with provider | 19.3 | 27.2 | 11.1 | 21.1 | 8.6 |
|  | Willing to share wearable data with provider | 32.7 | **76.8** | **68.1** | 0.0 | 0.0 |
| **Online health information and patient portal engagement** | Sought health information online | 86.4 | **96.7** | **78.3** | **95.5** | **60.3** |
|  | Messaged provider online | 67.4 | **91.8** | 26.4 | **88.5** | 17.7 |
|  | Viewed test results online | 74.6 | **97.2** | 43.2 | **95.2** | 23.5 |
|  | Scheduled appointment online | 68.9 | **91.4** | 46.6 | **81.9** | 25.4 |
|  | Accessed patient portal | 73.1 | **97.3** | 43.5 | **89.5** | 25.2 |
|  | Viewed clinical notes via portal | 58.9 | **86.7** | 21.3 | **75.0** | 10.2 |
| **Social media health engagement** | Visited social media (any) | 91.0 | **96.8** | **92.7** | **92.6** | **78.4** |
|  | Shared health information on social media | 34.8 | 49.3 | 20.1 | 36.2 | 18.4 |
|  | Interacted with similar-condition users | 28.0 | 42.2 | 12.8 | 30.3 | 10.7 |
|  | Watched health-related video | 71.1 | **82.5** | **70.8** | **75.1** | 47.2 |
| **Telehealth use** | Received telehealth care | 36.1 | **50.1** | 14.2 | 44.3 | 13.1 |

*Note.* Values are population-weighted percentages endorsing each indicator (response = 1) in the latent class analysis sample, estimated using the HINTS 7 final person weight and 50 jackknife replicate weights. Class-specific values are based on modal class assignment from the final four-class solution; unweighted Ns are shown in column headers. Values at or above 50% are shown in bold. All between-class comparisons were significant at p < .001 (Rao-Scott chi-square tests). Observed rates corresponded closely to the model-estimated item-response probabilities reported in Supplementary Table S2. Domain and indicator labels correspond to those in Supplementary Table S1.

**Supplementary Table S4.** *Characteristics of participants included in and excluded from the latent class analysis sample, and item-level missingness (N = 7,278)*

**Panel A. Participant characteristics by inclusion status**

| **Characteristic** | **Excluded (n = 1,474)** | **Included (n = 5,804)** | **p** |
| --- | --- | --- | --- |
| **Age, years** |  |  | **<.001** |
| 18–34 | 4.5 | 28.2 |  |
| 35–49 | 15.4 | 27.7 |  |
| 50–64 | 27.4 | 26.3 |  |
| 65–74 | 27.3 | 11.0 |  |
| ≥75 | 25.4 | 6.7 |  |
| **Sex** |  |  | **.694** |
| Female | 48.8 | 48.4 |  |
| Male | 50.8 | 50.8 |  |
| Unknown | 0.4 | 0.8 |  |
| **Education** |  |  | **<.001** |
| Less than high school | 15.3 | 4.9 |  |
| High school graduate | 26.9 | 20.5 |  |
| Some college | 34.8 | 39.1 |  |
| College graduate or higher | 23.1 | 35.5 |  |
| **Race and ethnicity** |  |  | **.020** |
| Hispanic | 18.8 | 17.4 |  |
| Non-Hispanic White | 56.9 | 61.1 |  |
| Non-Hispanic Black | 16.2 | 10.4 |  |
| NH American Indian / Alaska Native | 0.9 | 0.5 |  |
| Non-Hispanic Asian | 3.0 | 5.9 |  |
| NH Native Hawaiian / Pacific Islander | 0.2 | 0.3 |  |
| Non-Hispanic Multiple | 4.1 | 4.5 |  |
| **Household income** |  |  | **<.001** |
| <$20,000 | 24.5 | 13.7 |  |
| $20,000–34,999 | 15.4 | 11.0 |  |
| $35,000–49,999 | 10.8 | 11.7 |  |
| $50,000–74,999 | 16.8 | 15.4 |  |
| $75,000–99,999 | 9.8 | 12.8 |  |
| ≥$100,000 | 22.6 | 35.4 |  |
| **Smoking status** |  |  | **.278** |
| Never | 61.5 | 65.2 |  |
| Former | 26.2 | 22.8 |  |
| Current | 12.3 | 12.1 |  |
| **Marital status** |  |  | **<.001** |
| Married | 53.7 | 50.7 |  |
| Living with partner | 2.0 | 5.9 |  |
| Divorced | 9.7 | 6.2 |  |
| Widowed | 11.3 | 3.4 |  |
| Separated | 3.4 | 1.4 |  |
| Single | 19.8 | 32.4 |  |
| **Body mass index (kg/m²), mean (SD)** | **29.14 (7.25)** | **28.76 (7.05)** | **.380** |
| **PHQ-4 score, mean (SD)** | **1.99 (3.03)** | **2.42 (3.11)** | **.006** |

**Panel B. Item-level missingness among the 17 retained indicators**

| **Indicator** | **n missing** | **%** |
| --- | --- | --- |
| Willing to share wearable data with provider | 767 | 10.54 |
| Frequent wearable tracking | 754 | 10.36 |
| Viewed clinical notes via portal | 415 | 5.70 |
| Used a health or wellness app | 285 | 3.92 |
| Accessed patient portal | 221 | 3.04 |
| Received telehealth care | 183 | 2.51 |
| Shared device/app data with provider | 167 | 2.29 |
| Shared health information on social media | 138 | 1.90 |
| Interacted with similar-condition users | 107 | 1.47 |
| Watched health-related video | 100 | 1.37 |
| Viewed test results online | 97 | 1.33 |
| Uses wearable to track health or activity | 96 | 1.32 |
| Visited social media (any) | 95 | 1.31 |
| Scheduled appointment online | 92 | 1.26 |
| Messaged provider online | 91 | 1.25 |
| Sought health information online | 86 | 1.18 |
| Owns/uses smartwatch or wearable device | 58 | 0.80 |

*Note.* Panel A values are weighted column percentages unless otherwise indicated and are based on respondents with non-missing data for each characteristic. P values were obtained using design-adjusted Rao–Scott tests for categorical variables and survey-weighted Wald tests for continuous variables. Panel B reports unweighted counts of missing responses; nonapplicable responses generated by skip logic were coded as structural zeros and are not counted as missing. Willingness to share wearable data and frequency of wearable tracking together accounted for 1,521 of the 3,752 missing responses (40.5%). The primary latent class analysis sample was defined as respondents with complete data on all 18 candidate indicators; one portal-specific item assessing the viewing of test results was subsequently excluded because of strong redundancy and is therefore not presented in Panel B. PHQ-4 = Patient Health Questionnaire-4.

**Supplementary Table S5.** *Comparison of the primary complete-case analysis and the full information maximum likelihood sensitivity analysis*

|  | **Primary analysis (complete case)** | **Sensitivity analysis (FIML)** |
| --- | --- | --- |
| Latent class analysis sample, n | 5,804 | 7,278 |
| **Model-estimated class proportions, %** |  |  |
| Broad Multidomain Engagement | 32.0 | 29.2 |
| Wearable-Centered Engagement | 11.8 | 11.0 |
| Portal-Centered Engagement | 34.3 | 36.7 |
| Limited Engagement | 21.9 | 23.1 |
| Entropy | 0.911 | 0.914 |
| LMR-aLRT, 3 versus 4 classes | p = .014 | p = .003 |
| Modal class agreement with primary solution | — | 97.7% (κ = 0.97) |
| Regression sample, n | 4,930 | 5,745 |
| **Both guidelines met, aOR (95% CI)** |  |  |
| Broad Multidomain Engagement | 2.79 (1.81–4.30) | 2.91 (1.89–4.48) |
| Wearable-Centered Engagement | 2.13 (1.42–3.19) | 2.09 (1.39–3.14) |
| Portal-Centered Engagement | 1.36 (0.97–1.89) | 1.50 (1.13–2.00) |
| **Aerobic guideline met, aOR (95% CI)** |  |  |
| Broad Multidomain Engagement | 1.64 (1.17–2.29) | 1.85 (1.35–2.54) |
| Wearable-Centered Engagement | 1.38 (0.95–2.01) | 1.45 (1.02–2.08) |
| Portal-Centered Engagement | 0.87 (0.65–1.17) | 1.05 (0.83–1.33) |
| **Muscle-strengthening guideline met, aOR (95% CI)** |  |  |
| Broad Multidomain Engagement | 2.58 (1.85–3.60) | 2.60 (1.92–3.53) |
| Wearable-Centered Engagement | 1.64 (1.06–2.53) | 1.61 (1.12–2.32) |
| Portal-Centered Engagement | 1.58 (1.15–2.17) | 1.64 (1.24–2.17) |

*Note.* The sensitivity model was estimated on all HINTS 7 respondents using full information maximum likelihood for missing indicator data under a missing at random assumption; no respondent was missing all 17 indicators. Modal class agreement and Cohen’s κ were computed among the 5,804 respondents included in the primary latent class analysis. Limited Engagement is the reference class for all odds ratios. Adjusted models include age group, sex, education, race and ethnicity, household income, body mass index, PHQ-4 score, smoking status, and marital status. Information criteria are not compared across the two models because they were estimated on different numbers of observations. aOR = adjusted odds ratio; CI = confidence interval; FIML = full information maximum likelihood; LMR-aLRT = Lo-Mendell-Rubin adjusted likelihood ratio test; PHQ-4 = Patient Health Questionnaire-4.
